# Ankle muscle strength, joint hypermobility, and foot posture in children aged 5–10 years: A cross-sectional study

**DOI:** 10.1007/s00431-026-07313-7

**Published:** 2026-08-15

**Authors:** Carlos Martinez-Sebastian, Mari Carmen Carrasco De La Fuente, Gabriel Gijon-Nogueron, Alvaro Gomez-Carrion, Laura Ramos-Petersen, Angela M. F. Evans

**Affiliations:** 1 Department of Nursing and Podiatry, University of Malaga, Malaga, Spain; 2 PhD Program Biomedicina, Investigación Traslacional y Nuevas Tecnologías en Salud, University of Malaga, Malaga, Spain; 3 IBIMA, Plataforma BIONAND, Malaga, Spain; 4 Discipline of Podiatry, School of Allied Health, Human Services and Sport, La Trobe University, Melbourne, VIC Australia

**Keywords:** Paediatric foot, Joint hypermobility, Ankle strength, Foot posture index, Lower limb function

## Abstract

During childhood, foot posture, joint hypermobility, and foot and ankle strength may influence lower limb function. However, there is limited evidence analysing these variables together in the paediatric population. The study aims to analyse how foot posture, joint hypermobility, ankle mobility, and foot–ankle strength are related in children aged 5 to 10 years. A cross-sectional study was conducted in 196 healthy children aged between 5 and 10 years. Foot posture was assessed using the Foot Posture Index (FPI), general joint hypermobility using the Beighton score, and lower-limb–specific hypermobility using the Lower Limb Assessment Score (LLAS) and the Foot and Ankle Flexibility Index (FAFI). Ankle dorsiflexion was measured with the Lunge test, and isometric ankle strength was assessed using hand-held dynamometry. Descriptive analyses, correlations, and between-group comparisons were performed. No associations were found between foot posture, ankle dorsiflexion range of motion, or generalized joint hypermobility (Beighton score) and ankle muscle strength. In contrast, region-specific lower-limb hypermobility (LLAS and FAFI) showed only weak associations with ankle muscle strength. After adjustment for age, sex, and BMI-for-age z-score, only a limited number of associations remained statistically significant, and the observed effect sizes were small.

*Conclusions*: In healthy children, ankle muscle strength was not associated with foot posture, ankle dorsiflexion range of motion, or generalized joint hypermobility. Region-specific lower-limb hypermobility showed only weak associations with ankle muscle strength, most of which were attenuated after adjustment for age, sex, and BMI-for-age z-score.

**Table:** 

**What is Known:** *• Foot posture and joint hypermobility are common in childhood and may influence lower limb function.* *• Previous studies have analysed foot type, hypermobility or strength separately, with heterogenous findings.*	
**What is New:** *• Lower limb specific hypermobility (LLAS), but not Beighton score, was associated with ankle strength.* *• Hypermobile children showed greater relative ankle strength (BMI-normalised), suggesting threshold effects of joint laxity.*

**Supplementary Information:**

The online version contains supplementary material available at https://doi.org/10.1007/s00431-026-07313-7.

## Introduction

The foot and ankle play a fundamental role in child motor development, acting as the base of support of the body. During childhood, these structures undergo continuous bone, neuromuscular, and ligamentous maturation, allowing considerable adaptation [1–3]. The intrinsic and extrinsic musculature of the foot and ankle contributes to medial longitudinal arch support, load distribution during weight-bearing, and stability during gait; therefore, muscle strength is a key component of lower-limb function [4, 5].

Foot posture evolves substantially during childhood. Although physiological flatfoot is common in early years, the medial longitudinal arch typically develops progressively throughout the first decade of life [4, 5]. However, some children maintain pronated or supinated foot postures beyond this period [6]. The Foot Posture Index (FPI) provides a reliable and clinically applicable method for assessing foot posture in children [6], allowing objective classification without specialized equipment [7, 8].

Generalized joint hypermobility (GJH) is characterized by an increased range of motion in multiple joints, which is influenced by the characteristics of each child, such as age, sex, and ethnic background. Despite ongoing debate regarding the acceptability of the manoeuvres, tools and components of the assessment [9, 10], the Beighton score is the most commonly used measure to detect GJH and demonstrates good reliability [11]. To specifically assess the hypermobility of the lower limbs, Ferrari et al. [12] developed the Lower Limb Assessment Score (LLAS). Recently, Martínez et al. [13] validated a more specific instrument to assess joint hypermobility in the foot and ankle, a shortform of the LLAS, called the Foot and Ankle Flexibility Index (FAFI).

Although some studies have reported that children with flatter feet exhibit lower strength values in the ankle or the toe flexor muscles [14, 15], the evidence regarding muscle strength and generalized joint hypermobility remains inconsistent, with disparate results reported across paediatric and adult populations[16–18]. Most previous research has evaluated foot posture, mobility, or muscle strength independently, and limited evidence exists regarding the interaction between foot posture, region-specific hypermobility, ankle mobility, and ankle muscle strength during childhood.

Hand-held dynamometry provides a reliable method for quantifying isometric ankle strength in children aged five years or older, allowing standardized assessment of inversion, eversion, plantar flexion, and dorsiflexion strength [19, 20]. This approach is especially relevant in childhood, when ligamentous laxity and generalized joint hypermobility can alter the mechanical demands on the foot muscles and, potentially, their force-generating capacity.

The higher prevalence of hypermobility and pronated foot posture during early development suggests that muscle strength, joint mobility, and foot morphology may be interconnected [5, 21].

Our primary hypothesis was that children with greater hypermobility would have greater ankle strength due to increased neuromuscular effort. Therefore, the aim of this study is to explore the relationship between foot posture (FPI) generalized joint hypermobility (Beighton score), lower-limb–specific hypermobility (LLAS and FAFI), ankle mobility (lunge test), and isometric foot–ankle strength (dynamometry), in children aged 5 to 10 years.

## Materials and methods

### Ethical approval

The study fully complied with the principles of the Declaration of Helsinki for medical research in humans and received approval from the Ethics Committee of Universidad Católica San Antonio de Murcia (CE112104).

### Study design

This cross-sectional study was conducted according to the Strengthening the Reporting of Observational Studies in Epidemiology (STROBE) guidelines. Sample size was not based on a single primary outcome due to the exploratory nature of the study and the number of variables analysed. An a priori estimation indicated that a sample of approximately 123–193 participants would provide 80% power to detect small-to-moderate correlations (*r* = 0.20–0.25), while approximately 128 participants would be required for group comparisons assuming an effect size of d = 0.5. The final sample of 196 children was considered adequate for the planned analyses.

### Participants

Children aged 5–10 years were assessed between January and June 2022 at Colegio San Francisco de Asís (Lorca, Murcia, Spain).

Inclusion criteria were:

- age between 5 and 10 years;
- absence of foot pain at the time of assessment.

Exclusion criteria included congenital ankle abnormalities, cerebral palsy, previous foot or lower-limb surgery, and genetic, neurological, inflammatory, or muscular disorders.

### Procedure

Participants and their legal guardians were provided with detailed study information via electronic means, including objectives, procedures, and potential implications. Participation was voluntary and conditional upon obtaining written informed consent from the legal guardians, as well as assent from the children. The recruitment of the children was carried out among school grades that included children aged between 5 and 10 years. The classes were assessed sequentially, grade by grade. The participation rate of the measured children was 93% of the total school population. There were no missing data in the study.

Before starting the assessment, anthropometric measurements were obtained for all participants. Body mass index (BMI) was calculated as body mass (kg) divided by height squared (m^2^). In addition to absolute BMI values, BMI-for-age z-scores were calculated according to the World Health Organization 2007 growth reference for children and adolescents aged 5–19 years, taking into account participants' age and sex [22].

During the evaluation, participants were barefoot and wore appropriate sports attire. They were asked to maintain a relaxed posture while joint mobility measurements were performed within painless ranges.

Assessments were conducted by two examiners, both podiatry professionals with at least 7 years of experience in paediatric gait screening programs. All measures were performed bilaterally, on all participants.

The two examiners conducted hypermobility tests using the specified cut-off levels.

- Beighton Score ≥ 6/9 points [23].
- LLAS ≥ 7/12 points [12].
- FAFI ≥ 4/6 points [13].

The Beighton score was used to assess generalized joint hypermobility [23]. The LLAS is a tool specifically designed to evaluate hypermobility in the lower limbs, such as hips, knees, ankles, and feet. While the Beighton score gives an overall measure of joint laxity, the LLAS provides a more detailed assessment focused on the legs [12]. The FAFI, a shortform of the LLAS, is a specific instrument to assess joint hypermobility in the foot and ankle with 6 items [13].

Foot posture was assessed using the FPI, scoring six anatomical criteria on a scale from − 2 to + 2 to obtain a total score for each foot (ranging from − 12 for extreme supination to + 12 for extreme pronation) (Supplementary online material Fig. S1). This method has demonstrated good inter-examiner reliability in the paediatric population (Kw = 0.86) [24]. An FPI score of ≥  + 7 was used as the cut-off level for analysis, indicating a more pronated foot.

Ankle dorsiflexion range of motion was assessed using the weight-bearing lunge test. The maximum tibial inclination angle relative to vertical was recorded using a digital inclinometer while maintaining heel contact with the floor. This procedure has demonstrated excellent inter-examiner reliability in previous studies [25].

**Fig. 1:**
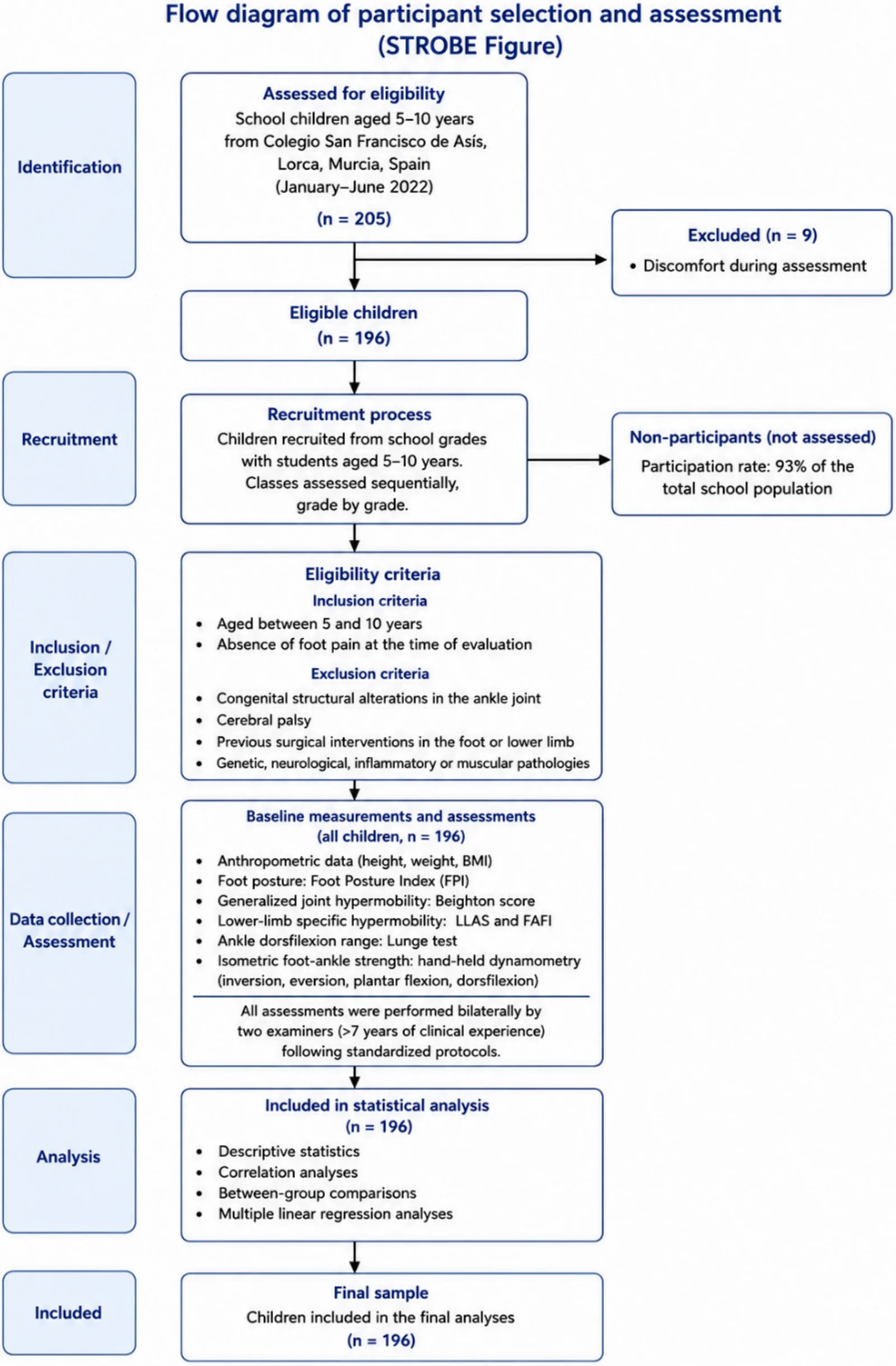
Flow diagram. Body mass index (BMI), Lower Limb Assessment score (LLAS)

Isometric ankle muscle strength was measured using a hand-held dynamometer (Lafayette Instrument Company, model 01160). Measurements were performed using the “make” test method, with participants seated, hips flexed, knees extended, and the back supported. Strength was assessed for inversion, eversion, plantar flexion, and dorsiflexion following standardized procedures [26–28]. Three maximal contractions lasting 3–5 s were performed for each movement, and the mean value was used for analysis. Previous studies have demonstrated high reliability of this protocol [29] (Fig. 1).

### Statistical analysis

Statistical analyses were performed using SPSS version 29 (IBM SPSS Statistics, 2022). Statistical significance was set at *p* < 0.05, and all tests were two-tailed.

Normality was assessed using the Kolmogorov–Smirnov test and data distribution inspection, while homogeneity of variance was evaluated using Levene’s test. Left and right limbs were analysed separately, considering each child as the unit of analysis.

Descriptive statistics were calculated for foot posture, hypermobility, lunge test, and strength variables. Results are presented as mean ± standard deviation and range.

Participants were classified according to FAFI criteria into hypermobile and non-hypermobile groups. Independent-samples *t*-tests were used to compare ankle strength according to hypermobility status and foot posture classification (neutral versus pronated). Supinated feet were excluded due to the small number of cases (*n* = 4). Effect sizes (Cohen’s *d*) and post hoc observed power were calculated for between-group comparisons.

Bivariate analyses were performed to determine the relationships between hypermobility and components of foot posture, ankle dorsiflexion, and ankle strength for both the left and right limbs.

Regarding strength, some studies adjust values relative to the individual’s body weight [30], while others indicate that height is also related to strength [31]. Based on these findings, strength was adjusted relative to weight and BMI.

Post hoc statistical power was calculated for all significant comparisons.

Agreement between the dichotomous classifications of lower-limb hypermobility (LLAS ≥ 7 and FAFI ≥ 4) was assessed using Cohen’s kappa coefficient.

Multiple linear regression analyses were conducted to investigate associations between ankle strength and hypermobility scores. Sixteen models were performed, with FAFI left, FAFI right, LLAS left, and LLAS right as dependent variables. Each model included one strength variable (inversion, eversion, dorsiflexion, or plantar flexion) as the predictor of interest, adjusted for age, sex, and BMI-for-age z-score. Regression assumptions were assessed through evaluation of linearity, homoscedasticity, and multicollinearity. Tolerance > 0.20 and variance inflation factor (VIF) < 5 were considered acceptable.

## Results

A total of 205 children were included, however, due to discomfort during assessment, 9 were excluded. Therefore, the final analysis included 196 children. The mean age was 7.6 years (range 5–10 years), with 59.2% girls and 40.8% boys. The mean body mass index (BMI) was 17.8 kg/m^2^ and standard deviation of 3.24. The BMI-for-age z-score was 0.85 ± 1.23 according to the WHO 2007 growth reference.

Table 1 presents descriptive statistics for foot posture, Lunge test, Beighton score, LLAS, FAFI, and strength expressed as a percentage of body weight (kg).

**Table 1:** Descriptive statistics of the study sample, including foot type, Lunge test, hypermobility assessments (Beighton, LLAS, and FAFI), and ankle strength (inversion, eversion, plantar flexion, and dorsiflexion)

*N* = 196	Mean	Standard deviation (SD)
BMI-for-Age Z-Score	0.85	1.23
Left FPI	3.9	2.8
Right FPI	3.8	2.7
Left Lunge Test (°)	50.4	6.5
Right Lunge Test (°)	50.3	6.5
Beighton Test	3.0	2.9
Left LLAS	5.5	3.5
Right LLAS	5.6	3.5
Left FAFI	3.4	2.2
Right FAFI	3.4	2.2
Left inversion strength (kg)	8.3	2.6
Right inversion strength (kg)	9.0	2.5
Left eversion strength (kg)	7.8	2.9
Right eversion strength (kg)	8.2	3.2
Left plantar flexion strength (kg)	15.4	7.6
Right plantar flexion strength (kg)	15.8	7.4
Left dorsiflexion strength (kg)	7.7	2.3
Right dorsiflexion strength (kg)	7.9	2.0

*SD* standard deviation, *FPI* foot posture index, *LLAS* lower limb assessment Score, *FAFI* foot and ankle flexibility index

No between-group differences were found when hypermobility was defined using the Beighton score (*p* > 0.05).

Small but significant correlations were observed between lower-limb hypermobility (LLAS and FAFI) and selected ankle strength variables (Table 2). Generalized joint hypermobility (Beighton score) was not associated with ankle strength. All significant correlations were weak (|r|≤ 0.24), indicating limited clinical relevance.

**Table 2:** Correlation matrix between study variables for right-limb hypermobility and components of foot posture, ankle dorsiflexion, and ankle strength, delineated by weight and BMI

			Weight				BMI			
*N* = 196			% Inversion Strength	% Eversion Strength	% Plantar Flexion Strength	% Dorsiflexion Strength	% Inversion Strength	% Eversion Strength	% Plantar Flexion Strength	% Dorsiflexion Strength
FPI	Left	r	.051	.022	.171*	.081	.001	-.016	.104	.033
		p	.480	.764	**.017**	.258	.991	.825	.146	.643
Lunge Test		r	.057	-.068	-.134	.128	-.144*	-.208**	-.216**	-.093
		p	.430	.346	.061	.074	**.044**	**.003**	**.002**	.193
Beighton Test		r	-.002	.003	-.002	.111	-.079	-.060	-.045	-.072
		p	.973	.962	.976	.120	.274	.405	.527	.317
LLAS		r	.160*	.060	-.005	.238**	-.074	-.114	-.131	-.084
		p	**.025**	.405	.945	**.001**	.303	.113	.067	.242
FAFI		r	.141*	.046	-.002	.197**	-.079	-.114	-.119	-.106
		p	**.049**	.522	.974	**.006**	.273	.112	.097	.139
FPI	Right	r	.071	.034	.136	.095	-,008	-.010	.068	.046
		p	.320	.637	.057	.186	.911	.889	.346	.518
Lunge Test		r	.069	.033	-.120	.155*	-.158*	-.118	-.208*	-.074
		p	.337	.645	.093	**.030**	**.027**	.099	**.003**	.303
Beighton Test		r	-.028	.034	-.071	.020	-.105	-.031	-.104	.022
		p	.694	.633	.320	.781	.142	.665	.146	.756
LLAS		r	.162*	.044	-.074	.165*	-.110	-.121	-.197*	.022
		p	**.024**	.539	.304	**.021**	.125	.090	**.006**	.764
FAFI		r	.135	.000	-.051	.134	-.108	-.142*	-.163*	.004
		p	.060	.997	.476	.062	.133	**.048**	**.023**	.955

*FPI* foot Posture index, *LLAS* lower limb assessment Score, *FAFI* foot and ankle flexibility index

* *p* < 0.05

** *p* < 0.01

There were no significant differences between the non-hypermobile and hypermobile foot and ankle groups (FAFI) for any of the strength-to-weight ratio or strength-to-BMI ratio variables on either the left or right side (Supplementary online material Table S1).

**Fig. 2:**
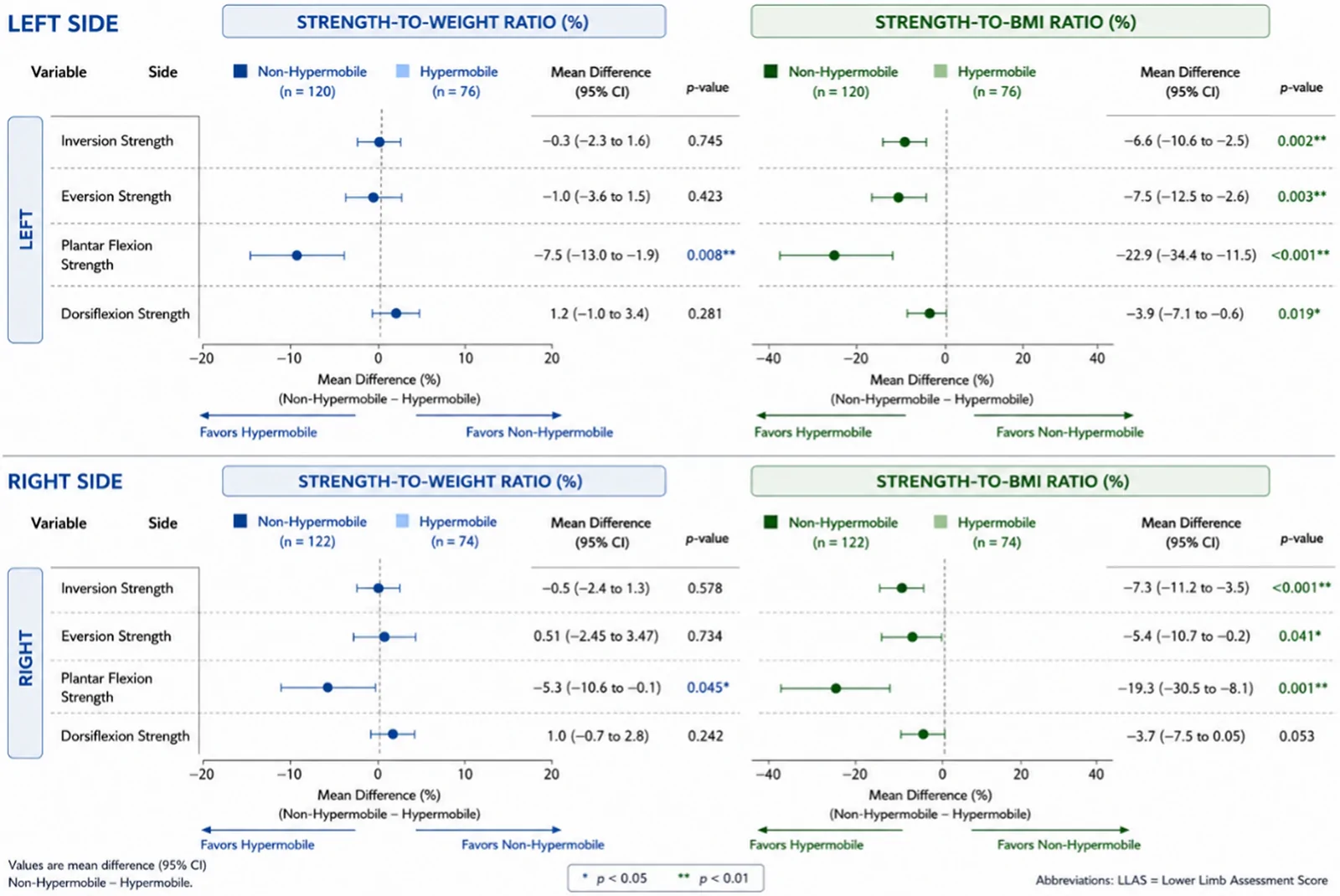
Comparison between participants with and without foot and ankle hypermobility across different strength variables in the right and left lower limbs (LLAS), expressed as strength-to-weight and strength-to-BMI ratios

For LLAS-defined hypermobility, plantar flexion strength-to-weight ratio was significantly greater in the hypermobile group on both sides, whereas no differences were observed for inversion, eversion, or dorsiflexion. When strength was normalized to BMI, hypermobile participants showed higher inversion, eversion, and plantar flexion strength bilaterally, while right dorsiflexion showed a borderline association (*p* = 0.053) (Fig. 2). Effect sizes (Cohen’s *d*) and observed power are presented in Supplementary Table S3.

For the strength-to-weight ratio, only left plantar flexion strength was significantly greater in pronated than in neutral feet (58.8 ± 22.6 vs. 51.7 ± 17.6; MD =  − 7.1; 95% CI: − 14.0 to − 0.2; *p* = 0.040). No significant differences were observed for inversion, eversion, or dorsiflexion strength on either side. Strength-to-BMI ratios likewise showed no statistically significant between-group differences, although right eversion strength (*p* = 0.050) and bilateral plantar flexion strength (left *p* = 0.080 and right *p* = 0.090) showed non-significant trends toward higher values in pronated feet (Table 3).

**Table 3:** Comparison between participants with neutral (FPI 0- + 6) and pronated (FPI > 6) feet for strength variables in the right and left feet, delineated by strength to weight and strength to BMI ratios

Variable	Side	Neutral Feet (*n* = 157) Mean ± SD	Pronated Feet (*n* = 35) Mean ± SD	Mean Difference (95% CI)	*p*-value	Neutral Feet (*n* = 156) Mean ± SD	Pronated Feet (*n* = 37) Mean ± SD	Mean Difference (95% CI)	*p*-value
		Strength-to-weight-ratio				Strength-to-BMI-ratio			
% Inversion Strength	Left	29.3 ± 6.8	29.6 ± 7.2	− 0.3 (− 2.9 to 2.1)	0.78	47.0 ± 14.0	48.1 ± 14.0	− 1.1 (− 6.3 to 4.0)	0.66
% Eversion Strength		27.2 ± 7.7	28.7 ± 11.0	− 1.4 (− 4.6 to 1.6)	0.34	43.8 ± 15.3	47.0 ± 20.5	− 3.2 (− 9.2 to 2.8)	0.29
% Plantar Flexion Strength-		51.7 ± 17.6	58.8 ± 22.6	− 7.1 (− 14.0 to − 0.2)	0.04*	84.6 ± 38.3	97.6 ± 45.1	− 12.9 (− 27.5 to 1.6)	0.08
% Dorsiflexion Strength		28.0 ± 8.6	27.5 ± 6.5	0.4 (− 2.6 to 3.5)	0.77	45.0 ± 11.3	46.3 ± 11	− 1.2 (− 5.4 to 2.9)	0.56
% Inversion Strength	Right	32.1 ± 6.5	32.7 ± 6.7	− 0.6 (− 2.9 to 1.7)	0.61	51.1 ± 13.8	53.6 ± 13.2	− 2.5 (− 7.4 to 2.4)	0.31
% Eversion Strength		28.5 ± 7.5	31.8 ± 17.5	− 3.36(− 7.0 to 0.3)	0.07	45.7 ± 15.8	52.2 ± 26.5	− 6.5 (− 13.1 to 0.1)	0.05
% Plantar Flexion Strength		53.3 ± 17.6	59.0 ± 21.2	− 5.67(− 12.3 to 0.9)	0.09	86.7 ± 38.6	99.0 ± 44.3	− 12.3 (− 26.7 to 1.9)	0.09
% Dorsiflexion Strength		28.3 ± 6.2	28.6 ± 6.3	− 0.2 (− 2.5 to 1.9)	0.80	44.0 ± 13.7	45.8 ± 11.4	− 1.7 (− 6.5 to 3.0)	0.46

Inter-rater reliability was excellent for FAFI (Kw = 0.89), Beighton (Kw = 0.80), LLAS (Kw = 0.78), lunge test (Kw = 0.88), and ankle strength (Kw = 0.75).

Agreement between LLAS and FAFI classifications was assessed using Cohen’s kappa coefficient. The agreement was poor for both lower limbs (left: κ = 0.169, *p* = 0.010; right: κ = 0.182, *p* = 0.008), indicating limited concordance between the two instruments in the classification of lower-limb hypermobility.

The results of the multiple linear regression analyses are summarized in Table 4. All models were adjusted for age, sex, and BMI-for-age z-scores.

**Table 4:** Multiple linear regression analyses for predictors of lower limb hypermobility

Outcome	Muscle strength	B (95% CI)	β	SE	p	Age β (p)	Sex β (p)	BMI-for-age Z-Score β (p)	Adjusted R^2^	VIF	Tolerance
**FAFI left**	**Inversion**	**0.204 (0.029 to 0.379)**	**0.236**	**0.089**	**0.022**	− 0.473 (< 0.001)	0.233 (0.001)	0.008 (0.910)	0.114	2.308	0.433
	Eversion	0.072 (− 0.069 to 0.213)	0.095	0.072	0.317	− 0.363 (< 0.001)	0.224 (0.002)	0.025 (0.723)	0.094	1.937	0.516
	**Dorsiflexion**	**0.193 (0.035 to 0.352)**	**0.200**	**0.080**	**0.017**	− 0.414 (< 0.001)	0.233 (0.001)	0.022 (0.744)	0.116	1.516	0.660
	**Plantarflexion**	**0.065 (0.002 to 0.127)**	**0.219**	**0.032**	**0.042**	− 0.466 (< 0.001)	0.232 (0.001)	0.012 (0.863)	0.109	2.511	0.394
**FAFI right**	Inversion	0.161 (− 0.027 to 0.350)	0.182	0.095	0.092	− 0.441 (< 0.001)	0.249 (< 0.001)	0.003 (0.963)	0.113	2.542	0.393
	Eversion	− 0.003 (− 0.123 to 0.117)	− 0.005	0.061	0.956	− 0.300 (0.001)	0.239 (0.001)	0.029 (0.674)	0.100	1.693	0.591
	Dorsiflexion	0.132 (− 0.083 to 0.346)	0.119	0.109	0.227	− 0.389 (< 0.001)	0.247 (0.001)	0.018 (0.800)	0.107	2.119	0.472
	Plantarflexion	0.040 (− 0.026 to 0.107)	0.134	0.034	0.232	− 0.409 (< 0.001)	0.251 (< 0.001)	0.019 (0.783)	0.106	2.720	0.360
**LLAS left**	Inversion	0.257 (− 0.016 to 0.531)	0.188	0.139	0.065	− 0.447 (< 0.001)	0.263 (< 0.001)	− 0.078 (0.259)	0.135	2.308	0.433
	Eversion	0.075 (− 0.145 to 0.295)	0.063	0.111	0.503	− 0.350 (< 0.001)	0.255 (< 0.001)	− 0.063 (0.362)	0.122	1.937	0.516
	Dorsiflexion	**0.302 (0.055 to 0.548)**	**0.197**	**0.125**	**0.017**	− 0.422 (< 0.001)	0.266 (< 0.001)	− 0.068 (0.313)	0.146	1.516	0.660
	Plantarflexion	0.079 (− 0.019 to 0.176)	0.169	0.049	0.112	− 0.437 (< 0.001)	0.262 (< 0.001)	− 0.074 (0.284)	0.131	2.511	0.398
**LLAS right**	Inversion	0.201 (− 0.089 to 0.492)	0.145	0.147	0.173	− 0.439 (< 0.001)	0.251 (< 0.001)	− 0.080 (0.247)	0.138	2.542	0.393
	Eversion	0.023 (− 0.162 to 0.207)	0.021	0.094	0.810	− 0.341 (< 0.001)	0.243 (0.001)	− 0.062 (0.366)	0.129	1.693	0.591
	Dorsiflexion	0.161 (− 0.169 to 0.491)	0.093	0.167	0.338	− 0.396 (< 0.001)	0.250 (< 0.001)	− 0.069 (0.316)	0.133	2.119	0.472
	Plantarflexion	0.029 (− 0.074 to 0.132)	0.061	0.052	0.583	− 0.377 (0.001)	0.249 (< 0.001)	− 0.064 (0.347)	0.131	2.720	0.368

*B* unstandardized regression coefficient, *SE* standard error, *β* standardized regression coefficient, *CI* confidence interval, *VIF* variance inflation factor, *BMI* body mass index, *FAFI* foot and ankle flexibility index, *LLAS* lower limb assessment score

For the FAFI outcomes, greater inversion strength was independently associated with higher FAFI scores on the left side (B = 0.204, 95% CI 0.029 to 0.379; β = 0.236; *p* = 0.022). Greater dorsiflexion strength (B = 0.193, 95% CI 0.035 to 0.352; β = 0.200; *p* = 0.017) and plantarflexion strength (B = 0.065, 95% CI 0.002 to 0.127; β = 0.219; *p* = 0.042) were also independently associated with higher FAFI left scores, whereas eversion strength was not (*p* = 0.317). For FAFI right, none of the muscle strength variables were independently associated with the outcome after controlling for age, sex, and BMI-for-age z-scores.

For the LLAS outcomes, dorsiflexion strength was the only muscle strength variable independently associated with LLAS left (B = 0.302, 95% CI 0.055 to 0.548; β = 0.197; *p* = 0.017). No significant independent associations were observed for inversion, eversion, or plantarflexion strength. Similarly, none of the muscle strength variables were significantly associated with LLAS right after adjustment for the covariates.

Across all models, increasing age was independently associated with lower FAFI and LLAS scores, whereas females had higher scores than males (all *p* ≤ 0.001). BMI-for-age z-score was not a significant predictor. Adjusted *R*2 ranged from 0.094 to 0.146 for FAFI and from 0.122 to 0.138 for LLAS. No problematic multicollinearity was detected (all VIF < 3; tolerance > 0.36).

## Discussion

This study examined the relationships between foot posture, joint hypermobility, ankle dorsiflexion, and isometric ankle strength in children aged 5–10 years.

Normative reference values for strength and flexibility show a progressive increase in muscle strength with age, particularly during childhood and adolescence, which correlates with height and body mass [31].

Ankle dorsiflexion plays a key role in gait dynamics, allowing the foot to clear the ground and facilitating proper foot placement during stance [32]. The interaction between joint mobility and ankle strength has been studied in young adults, showing that individuals with greater dorsiflexion range also exhibit higher dorsiflexor strength, with a slight association to first-toe flexor strength [33]. In children with idiopathic toe-walking, a reduced dorsiflexion range has been reported along with decreased strength in the triceps surae and tibialis anterior muscles [34]. However, in our paediatric sample, no significant association was found between ankle dorsiflexion range and isometric ankle strength, suggesting that this relationship may not be present in early childhood or may be influenced by developmental factors.

Given the prepubertal age range of the participants, sex-related differences in muscle strength were not considered in the study design. Therefore, strength data from boys and girls were pooled for analysis, in line with previous evidence suggesting that sex differences in strength assessed using hand-held dynamometry are not typically evident before puberty [35].

Studies examining the relationship between muscle strength and joint hypermobility commonly use the Beighton score for assessment, which is limited for evaluating the lower limb, as it only includes the knee. In this limited context, Pranay et al. [18] evaluated knee extensor strength in young adults and found no significant differences in muscle strength between females with and without hypermobility, and in males, a lower strength was observed only in the right knee of hypermobile individuals.

In the paediatric population, one study [36] analysed ankle dorsiflexor and plantar flexor strength in children with and without generalized joint hypermobility, and reported decreased isometric lower-limb strength in the hypermobile group. In comparison with these findings, our results showed no correlation between ankle strength and Beighton scores, which assesses generalised joint ranges, but does not examine the ankle.

Unlike the Beighton score, lower-limb hypermobility assessed using the FAFI and LLAS showed weak associations with ankle muscle strength. Children classified as hypermobile, particularly according to the LLAS, demonstrated greater ankle strength than non-hypermobile children in some unadjusted comparisons. Although hypermobility has often been associated with muscle weakness in symptomatic populations, findings in healthy children remain inconsistent and may reflect neuromuscular adaptations that enhance dynamic joint stability. To our knowledge, no previous studies have examined the relationship between region-specific lower-limb hypermobility (FAFI or LLAS) and ankle muscle strength in children. In our study, most associations disappeared after adjustment for age, sex, and BMI-for-age z-score. Only inversion, dorsiflexion, and plantarflexion strength remained independently associated with left FAFI, and only dorsiflexion with left LLAS. However, the regression coefficients were small and the models explained only a modest proportion of the variance (adjusted *R*2 = 0.09–0.15), indicating limited clinical relevance.

The poor agreement between LLAS and FAFI (κ = 0.169–0.182) indicates that these instruments are not interchangeable and may assess different aspects of lower-limb hypermobility, which may explain their differing associations with ankle strength.

The interpretation of the findings depended on the analytical approach. Correlation analyses identified only weak linear associations, whereas group comparisons detected differences between clinically defined hypermobile and non-hypermobile children. Most of these differences disappeared after adjustment for age, sex, and BMI-for-age z-score, suggesting that growth-related factors may partly explain the observed associations. A threshold effect cannot be excluded but should be interpreted cautiously and confirmed in longitudinal studies.

Although several associations reached statistical significance, their clinical relevance appears limited. Regression coefficients were small, and the models explained only a modest proportion of the variance (adjusted *R*2 = 0.09–0.15), indicating that ankle muscle strength is only one of several factors associated with lower-limb hypermobility. Age was the strongest predictor, female sex was associated with higher LLAS and FAFI scores, whereas BMI-for-age z-score was not independently associated with either outcome.

FAFI showed more consistent independent associations than LLAS in the multivariable models, whereas LLAS identified more differences in the unadjusted analyses. The greater anatomical specificity of the FAFI may explain its stronger associations with ankle strength. However, effect sizes were small, indicating limited clinical relevance.

An important methodological finding was that significant differences between hypermobile and non-hypermobile participants were observed mainly when ankle strength was normalised to BMI, whereas fewer differences were detected when strength was normalised only to body weight. This indicates that the method used to express muscle strength may influence the observed associations. However, BMI-for-age z-score was not an independent predictor of either LLAS or FAFI in the multivariable regression analyses, suggesting that the observed differences are more likely related to the scaling effect introduced by anthropometric normalisation than to BMI itself. Therefore, the greater relative strength observed in children with lower-limb hypermobility is unlikely to reflect an independent effect of hypermobility itself, but rather the influence of anthropometric scaling and normal growth-related characteristics on the expression of muscle strength.

While hypermobility is common and has previously been associated with alterations in muscle strength, foot type and ankle strength remain poorly investigated in paediatric populations.

In the paediatric population, three previous studies have examined ankle and toe-grip strength in relation to foot type. Tashiro et al. [37] investigated toe flexor strength in different foot types classified by footprint and found that toe-grip strength was associated with foot posture, and that flat feet showed lower toe-grip strength compared to those with normal foot posture. Kim et al. [38] used a small paediatric sample to relate foot posture via FPI, with ankle inverter and evertor strength and to measure calf muscle thickness. Children with more pronated feet showed significantly lower inverter and evertor strength, as well as reduced thickness of the posterior tibial and peroneal muscles. Hashimoto et al.[39] showed that improving toe-grip strength can contribute to increasing the foot arch in adolescents. Additionally, toe-grip strength was observed to be related to foot posture in this population. In contrast, our findings differ from these previous studies, as significant differences were not observed in ankle strength between pronated and neutral-footed children.

FPI was not significantly associated with isometric ankle strength in most comparisons. These findings suggest that, in children aged 5 to 10 years, the distinction between neutral and pronated foot posture does not appear to meaningfully influence ankle force-generating capacity. One possible explanation is that static foot posture and muscle strength represent different aspects of lower-limb function. The FPI evaluates foot alignment during quiet standing, whereas ankle strength was assessed during isolated maximal isometric contractions. Consequently, a more pronated foot posture does not necessarily imply impaired force-generating capacity, particularly in healthy children without pain or functional limitations. It is also possible that neuromuscular adaptations compensate for variations in foot posture during normal development, allowing children with different foot postures to achieve similar levels of ankle muscle strength.

The absence of a clear relationship may also reflect the dynamic nature of foot development during childhood. Between 5 and 10 years of age, substantial changes occur in foot morphology and neuromuscular control, and static foot posture may therefore be a poor surrogate for muscular function. This is consistent with longitudinal evidence showing that foot posture continues to mature throughout childhood, with considerable inter-individual variability in arch development [5].

There are limitations to our study. It is a cross-sectional design, which prevents establishing causal relationships between the factors evaluated. In addition, the sample was mainly asymptomatic Caucasian children aged 5 to 10 years. Participants were recruited from a single school through a convenience-based, consent-dependent sampling approach. Although the participation rate was high, selection bias cannot be excluded, as non-participants and children excluded due to discomfort were not characterized. Therefore, children who participated may differ from those who did not, potentially limiting the generalizability of the findings.

Future studies should include larger and more diverse paediatric populations and use longitudinal designs to clarify the evolution of hypermobility, ankle strength, BMI, and foot posture during growth, as well as their relationship with functional outcomes.

## Conclusion

In healthy children, region-specific lower-limb hypermobility showed only weak associations with ankle muscle strength, whereas generalized joint hypermobility assessed using the Beighton score was not associated with ankle strength. After adjustment for age, sex, and BMI-for-age z-score, only a small number of associations remained, and their effect sizes were modest, indicating that ankle muscle strength explains only a limited proportion of lower-limb hypermobility. No association was observed between ankle dorsiflexion range of motion and ankle muscle strength, nor were differences in ankle muscle strength identified according to foot posture as assessed by the Foot Posture Index (FPI). These findings suggest that ankle muscle strength alone has limited clinical utility as an indicator of lower-limb hypermobility in healthy children.

These findings contribute to a better understanding of foot and ankle development during childhood and highlight the importance of accounting for growth-related anthropometric variation when investigating the relationship between ankle muscle strength and lower-limb hypermobility.

## Supplementary Information

Below is the link to the electronic supplementary material.

Supplementary file1 (DOCX 18.3 KB)

Supplementary file2 (PDF 885 KB)

Supplementary file3 (DOCX 18.5 KB)

Supplementary file4 (DOCX 19.2 KB)

## Data Availability

The datasets generated and/or analyzed during the current study are available from the corresponding author on reasonable request.

## Abbreviations

BMI: Body Mass Index
CI: Confidence Interval
FAFI: Foot and Ankle Flexibility Index
FPI: Foot Posture Index
GJH: Generalized Joint Hypermobility
LLAS: Lower Limb Assessment Score
MD: Mean Difference
SPSS: Statistical Package for the Social Sciences
STROBE: Strengthening the Reporting of Observational Studies in Epidemiology
