# Supplementary material for: Ankle muscle strength, joint hypermobility, and foot posture in children aged 5–10 years: A cross-sectional study
Source: Eur J Pediatr. 2026 Aug 15;185(9):668. doi: 10.1007/s00431-026-07313-7 (PMC13477486; doi:10.1007/s00431-026-07313-7)
Supplement: Supplementary file 1 [file 431_2026_7313_MOESM2_ESM.pdf]

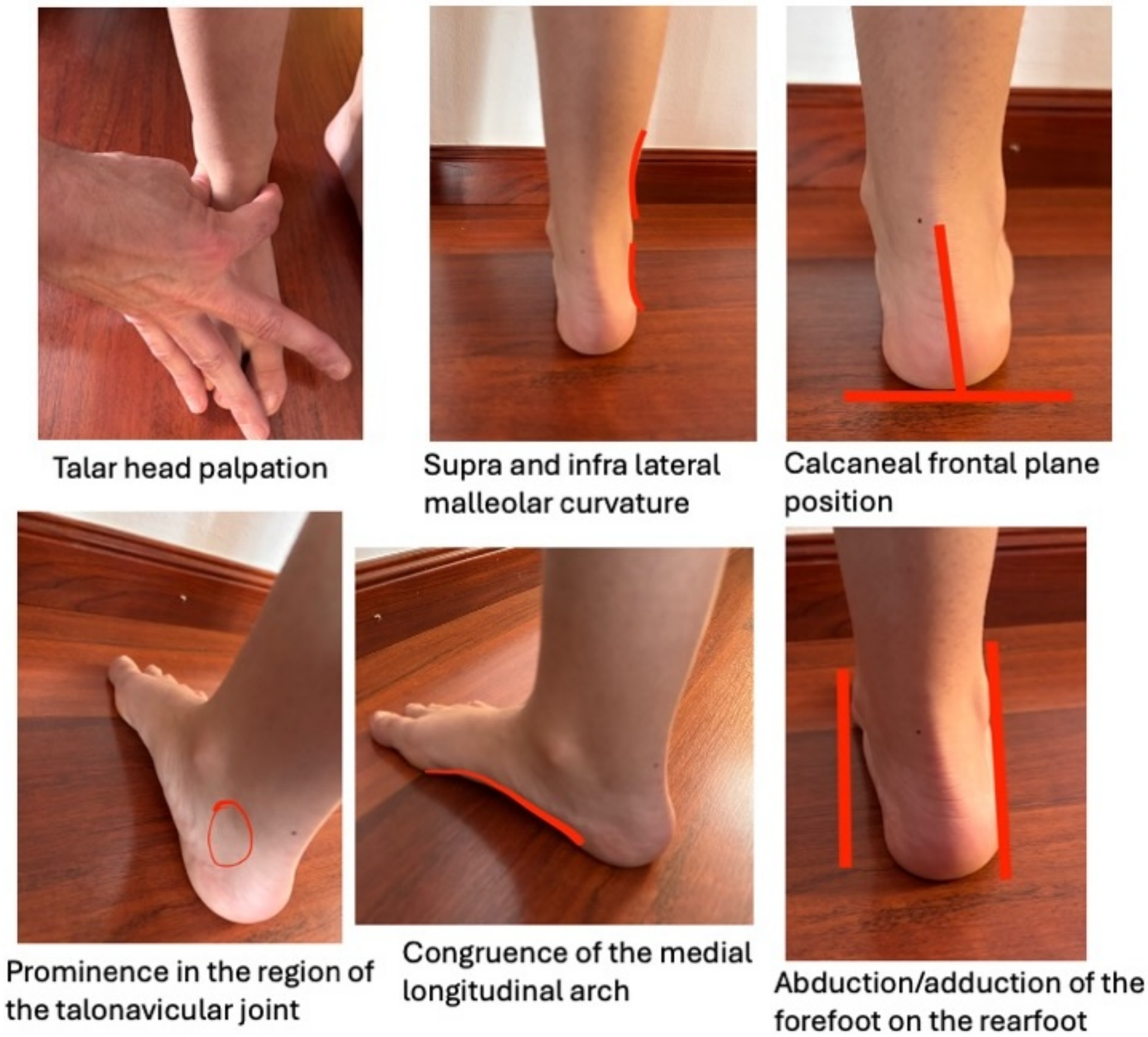

Supplementary online material Figure S1. 6 items of FPI.
