# Supplementary material for: Ankle muscle strength, joint hypermobility, and foot posture in children aged 5–10 years: A cross-sectional study
Source: Eur J Pediatr. 2026 Aug 15;185(9):668. doi: 10.1007/s00431-026-07313-7 (PMC13477486; doi:10.1007/s00431-026-07313-7)
Supplement: Supplementary file 2 [file 431_2026_7313_MOESM1_ESM.docx]

| **Variable** | **Side** | **Non-Hypermobile (n = 120) Mean ± SD** | **Hypermobile (n = 76) Mean ± SD** | **Mean Difference (95% CI)** | **p-value** | **Cohen's** | **Non-Hypermobile (n = 120) Mean ± SD** | **Hypermobile (n = 76) Mean ± SD** | **Mean Difference (95% CI)** | **p-value** | **Cohen's d** |
| --- | --- | --- | --- | --- | --- | --- | --- | --- | --- | --- | --- |
|  |  | Strength-to-weight-ratio | | | |  | Strength-to-BMI-ratio | | | |  |
| % Inversion Strength | Left | 29.1 ± 6.6 | 29.5 ± 7.1 | −0.3 (−2.3 to 1.6) | 0.745 | −0.17 | 44.5 ± 12.7 | 51.1 ± 14.7 | −6.6 (−10.6 to −2.5) | **0.002**** | 0.80 |
| % Eversion Strength |  | 26.9 ± 7.4 | 28.0 ± 9.7 | −1.0 (−3.6 to 1.5) | 0.423 | −0.08 | 41.2 ± 13.6 | 48.8 ± 18.9 | -7.5 (−12.5 to−2.6) | **0.003**** | 0.23 |
| % Plantar Flexion Strength- |  | 49.9 ± 16.7 | 57.4 ± 20.6 | −7.5 (−13.0 to −1.9) | **0.008**** | 0.12 | 77.8 ± 35.0 | 100.7 ± 42.2 | -22.9 (−34.4 to−11.5) | **<0.001**** | 0.51 |
| % Dorsiflexion Strength |  | 28.3 ± 9.1 | 27.1 ± 6.6 | 1.2 (−1.0 to 3.4) | 0.281 | −0.40 | 43.6 ± 10.8 | 47.5 ± 11.8 | −3.9 (−7.1 to −0.6) | **0.019*** | 0.48 |
| **Variable** | **Side** | **Non-Hypermobile (n = 122) Mean ± SD** | **Hypermobile (n = 74) Mean ± SD** | **Mean Difference (95% CI)** | **p-value** | **Cohen's** | **Non-Hypermobile (n = 122) Mean ± SD** | **Hypermobile (n = 74) Mean ± SD** | **Mean Difference (95% CI)** | **p-value** | **Cohen's d** |
| % Inversion Strength | Right | 31.9 ± 6.4 | 32.4 ± 6.7 | -0.5 (−2.4 to1.3) | 0.578 | −0.21 | 48.6 ± 12.8 | 56.0 ± 13.8 | −7.3 (−11.2 to −3.5) | **< 0.001**** | 0.48 |
| % Eversion Strength |  | 29.28 ± 11.44 | 28.77 ± 8.05 | 0.51 (−2.45 to3.47) | 0.734 | 0.05 | 44.7 ± 18.9 | 50.2 ± 17.0 | −5.4 (−10.7 to −0.2) | **0.041*** | 0.10 |
| % Plantar Flexion Strength |  | 52.2 ± 17.4 | 57.6 ± 19.3 | -5.3 (−10.6 to−0.1) | **0.045*** | 0.27 | 81.5 ± 37.2 | 100.8 ± 40.9 | −19.3 (−30.5 to −8.1) | **0.001**** | −0.15 |
| % Dorsiflexion Strength |  | 28.7 ± 6.1 | 27.6 ± 6.4 | 1.0 (−0.7 to 2.8) | 0.242 | −0.21 | 42.8 ± 13.7 | 46.6 ± 12.3 | −3.7 (−7.5 to 0.05) | 0.053 | −0.30 |

**Abbreviations:** FPI= Foot Posture Index;

* p <0.05

** p < 0.01

(Supplementary online material Table S2). Comparison between participants with and without foot and ankle hypermobility for various variables in the right and left limb (LLAS), delineated by strength to weight and strength to BMI ratios.
