# Supplementary material for: Ankle muscle strength, joint hypermobility, and foot posture in children aged 5–10 years: A cross-sectional study
Source: Eur J Pediatr. 2026 Aug 15;185(9):668. doi: 10.1007/s00431-026-07313-7 (PMC13477486; doi:10.1007/s00431-026-07313-7)
Supplement: Supplementary file 4 [file 431_2026_7313_MOESM4_ESM.docx]

Supplementary Table 3. Effect sizes and observed power for ankle muscle strength differences between hypermobile and non-hypermobile groups

| **Instrument** | **Strength normalization** | **Variable** | **Side** | **n (Non-Hypermobile)** | **n (Hypermobile)** | **Cohen's d** | **Observed power (1−β)** |
| --- | --- | --- | --- | --- | --- | --- | --- |
| LLAS | Strength-to-weight ratio | Inversion | Left | 120 | 76 | 0.18 | 0.231 |
| LLAS | Strength-to-weight ratio | Eversion | Left | 120 | 76 | 0.08 | 0.084 |
| LLAS | Strength-to-weight ratio | Plantar flexion | Left | 120 | 76 | 0.10 | 0.104 |
| LLAS | Strength-to-weight ratio | Dorsiflexion | Left | 120 | 76 | 0.41 | 0.795 |
| LLAS | Strength-to-weight ratio | Inversion | Right | 122 | 74 | 0.25 | 0.393 |
| LLAS | Strength-to-weight ratio | Eversion | Right | 122 | 74 | 0.02 | 0.052 |
| LLAS | Strength-to-weight ratio | Plantar flexion | Right | 122 | 74 | 0.32 | 0.580 |
| LLAS | Strength-to-weight ratio | Dorsiflexion | Right | 122 | 74 | 0.29 | 0.499 |
| LLAS | Strength-to-BMI ratio | Inversion | Left | 120 | 76 | 0.23 | 0.345 |
| LLAS | Strength-to-BMI ratio | Eversion | Left | 120 | 76 | 0.24 | 0.371 |
| LLAS | Strength-to-BMI ratio | Plantar flexion | Left | 120 | 76 | 0.34 | 0.636 |
| LLAS | Strength-to-BMI ratio | Dorsiflexion | Left | 120 | 76 | 0.21 | 0.297 |
| LLAS | Strength-to-BMI ratio | Inversion | Right | 122 | 74 | 0.31 | 0.553 |
| LLAS | Strength-to-BMI ratio | Eversion | Right | 122 | 74 | 0.39 | 0.750 |
| LLAS | Strength-to-BMI ratio | Plantar flexion | Right | 122 | 74 | 0.57 | 0.971 |
| LLAS | Strength-to-BMI ratio | Dorsiflexion | Right | 122 | 74 | 0.02 | 0.052 |
| FAFI | Strength-to-weight ratio | Inversion | Left | 99 | 97 | 0.20 | 0.286 |
| FAFI | Strength-to-weight ratio | Eversion | Left | 99 | 97 | 0.14 | 0.164 |
| FAFI | Strength-to-weight ratio | Plantar flexion | Left | 99 | 97 | 0.11 | 0.119 |
| FAFI | Strength-to-weight ratio | Dorsiflexion | Left | 99 | 97 | 0.24 | 0.387 |
| FAFI | Strength-to-weight ratio | Inversion | Right | 98 | 98 | 0.23 | 0.360 |
| FAFI | Strength-to-weight ratio | Eversion | Right | 98 | 98 | 0.01 | 0.051 |
| FAFI | Strength-to-weight ratio | Plantar flexion | Right | 98 | 98 | 0.17 | 0.220 |
| FAFI | Strength-to-weight ratio | Dorsiflexion | Right | 98 | 98 | 0.24 | 0.387 |
| FAFI | Strength-to-BMI ratio | Inversion | Left | 99 | 97 | 0.21 | 0.310 |
| FAFI | Strength-to-BMI ratio | Eversion | Left | 99 | 97 | 0.29 | 0.524 |
| FAFI | Strength-to-BMI ratio | Plantar flexion | Left | 99 | 97 | 0.37 | 0.731 |
| FAFI | Strength-to-BMI ratio | Dorsiflexion | Left | 99 | 97 | 0.20 | 0.286 |
| FAFI | Strength-to-BMI ratio | Inversion | Right | 98 | 98 | 0.10 | 0.107 |
| FAFI | Strength-to-BMI ratio | Eversion | Right | 98 | 98 | 0.20 | 0.286 |
| FAFI | Strength-to-BMI ratio | Plantar flexion | Right | 98 | 98 | 0.30 | 0.552 |
| FAFI | Strength-to-BMI ratio | Dorsiflexion | Right | 98 | 98 | 0.02 | 0.052 |

Abbreviations: LLAS, Lower Limb Assessment Score; FAFI, Foot and Ankle Flexibility Index. Cohen’s d represents the standardized mean difference between non-hypermobile and hypermobile groups. Observed power (1−β) was calculated post hoc for each comparison.
